# Biogenic Selenium Nanoparticles Synthesized with Alginate Oligosaccharides Alleviate Heat Stress-Induced Oxidative Damage to Organs in Broilers through Activating Nrf2-Mediated Anti-Oxidation and Anti-Ferroptosis Pathways

**DOI:** 10.3390/antiox12111973

**Published:** 2023-11-06

**Authors:** Xue-Qing Ye, Yan-Ru Zhu, Yu-Ying Yang, Sheng-Jian Qiu, Wen-Chao Liu

**Affiliations:** 1 Department of Animal Science, College of Coastal Agricultural Sciences, Guangdong Ocean University, Zhanjiang 524088, China; yexueqing1@stu.gdou.edu.cn (X.-Q.Y.); yangyuying@stu.gdou.edu.cn (Y.-Y.Y.); qiushengjian@stu.gdou.edu.cn (S.-J.Q.); 2 Department of Animal Nutrition and Environmental Health, College of Animal Science and Technology, Northwest A&F University, Xi’an 712100, China; zhuyanru@nwafu.edu.cn

**Keywords:** antioxidation, broiler, ferroptosis, heat stress, organ health, selenium nanoparticles

## Abstract

Selenium (Se) is an essential trace element for maintaining health due to its ideal antioxidant properties. We previously prepared a new type of biogenic selenium nanoparticles based on alginate oligosaccharides (SeNPs-AOS), and this study aimed to investigate the protective effects of SeNPs-AOS (Se particle size = 80 nm, Se content = 8%) on organ health in broilers challenged with HS. A total of 192 21-day-old Arbor Acres broilers were randomly divided into four groups according to a 2 × 2 experimental design, including a thermoneutral zone group (TN, raised under 23 ± 1.5 °C); TN + SeNPs-AOS group (TN group supplemented 5 mg/kg SeNPS-AOS); HS group (HS, raised under 33 ± 2 °C for 10 h/day); and HS + SeNPs-AOS group (HS group supplemented 5 mg/kg SeNPS-AOS). There were six replicates in each group (eight broilers per replicate). The results showed that SeNPs-AOS improved the splenic histomorphology, enhanced the activity of catalase (CAT) and glutathione peroxidase (GSH-Px) of the spleen, as well as upregulating the splenic mRNA expression of antioxidant-related genes in broilers under HS. In addition, SeNPs-AOS reversed the pathological changes in bursa caused by HS increased the activity of GST, GSH-Px, and CAT and upregulated the mRNA expression of *Nrf2* and antioxidant-related genes in the bursa of heat-stressed broilers. In addition, dietary SeNPs-AOS improved the hepatic damage, increased the activity of GSH-Px in the liver, and upregulated the mRNA expression of antioxidant-related genes while downregulating the *Keap1* gene expression of the liver in broilers during HS. Moreover, dietary SeNPs-AOS upregulated the anti-ferroptosis-related genes expression of liver in broilers under HS. In conclusion, dietary SeNPs-AOS could relieve HS-induced oxidative damage to the spleen, bursa of Fabricius and liver in broilers by upregulating the Nrf2-mediated antioxidant gene expression and SeNPs-AOS could also upregulate the expression of hepatic Nrf2-related anti-ferroptosis genes in heat-stressed broilers. These findings are beneficial for the development of new nano-antioxidants in broilers.

## 1. Introduction

With the rise of global temperature and high-density feeding, heat stress (HS) has become the primary environmental stressor in broiler production, which has deleterious impacts on broiler productivity and health [1]. It is already clear that HS results in reduced feed intake, growth restriction, and metabolic disorders in broilers [2,3]. Meanwhile, multiple studies have found that HS-induced oxidative stress in organs, leading to organ injury and dysfunction, which is also an important reason for HS causing production losses in broilers [4,5,6]. The spleen and bursa of Fabricius are essential immune organs for broilers [7]. Besides, liver plays a pivotal role in digestion and metabolic homeostasis [8]. However, HS overproduces reactive oxygen species (ROS) and destroys the antioxidant system, which causes oxidative damage to organs [9]. It has been suggested that HS resulted in atrophy of spleen and bursa, and varying degrees damage of liver, which could be attributed to oxidative stress [10,11]. Furthermore, ferroptosis is commonly accompanied by oxidative stress, and it is an important manifestation of organ oxidative damage [12,13]. Glutathione is crucial for regulating ferroptosis, and the liver is the main biosynthesis factory for glutathione [14]. Therefore, oxidative stress is a key factor that causes organ injuries in broilers subjected to HS, and hepatocytes are more prone to ferroptosis under oxidative stress.

Selenium (Se) is one of the essential trace elements for maintaining normal physiological functions [15]. As a trace element with antioxidant effects, Se plays an indispensable role in the redox balance [16]. Studies have shown that dietary Se deficiency reduced the expression of selenoprotein genes [17]. For instance, Se deficiency sharply decreased the expression of the *GPX4* gene and induced oxidative stress in organs [18]. In addition, dietary Se deficiency has been reported to increase the risk of metabolic diseases, resulting in metabolic disorders in the body [19]. As a new form of Se supplement, selenium nanoparticles (SeNPs) can maximize the beneficial effects of Se because of its high surface activity and nanoscale effect [20]. Compared with inorganic selenium, SeNPs have a higher absorption rate, higher biological titer, and lower biological toxicity [15]. Moreover, SeNPs are cheaper and have a higher stability compared to organic selenium [21]. However, ordinary SeNPs are unstable in stored and easy to form precipitation, which affects their application in practice; biogenic SeNPs may be a strategy to improve this shortcoming [22]. It is interesting that, with the development of modern green nanobiotechnology, emerging studies have synthesized biogenic SeNPs based on natural polymers, and discovered their behaviors in protecting intestinal antioxidant system of animals [23]. In addition, biogenic SeNPs have been reported to activate the Nrf2-antioxidant pathway, effectively strengthening the antioxidant capacity and relieving the oxidative stress in organs [24].

Alginate oligosaccharides (AOS) are natural polymers from marine sources with various biological activities [25,26]. In our previous study, we synthesized a novel type of biogenic selenium nanoparticle using alginate oligosaccharides (SeNPs-AOS) and found that the Se particle size and the Se content are 80 nm and 8%, respectively. We hypothesized that the preparation of biogenic SeNPs with AOS possesses both Se and AOS bioactivity [27]. In addition, the SeNPs-AOS have a nano-size effect and can increase the bioavailability of Se, thereby improving the antioxidant properties. Also, in the earlier study, we found that SeNPs-AOS promoted the growth performance, such as average daily gain (ADG) of heat-stressed broilers at 22–28 days of age [27]. Unfortunately, no studies have been reported about the protective effects of biogenic SeNPs synthesized with AOS on organ health in animals under HS. Therefore, the present study aimed to evaluate whether SeNPs-AOS can alleviate HS-induced organ oxidative damage in broilers.

## 2. Materials and Methods

### 2.1. Synthesis of SeNPs-AOS

In our study, the synthesis of SeNPs-AOS used AOS as a polymer template, followed by the reduction of sodium selenite (Na_2_SeO_3_) by ascorbic acid (Vc). AOS was provided by Qingdao Bozhi Huili Biotechnology Co., Ltd. (Qingdao, China). Na_2_SeO_3_ was produced by SIGMA Co., Ltd. (Shanghai, China). Vc was provided by Xilong Science Co., Ltd. (Shantou, China). Based on the analysis results of high-performance liquid chromatography (HPLC), the oligosaccharides are mainly composed of glucosamine (GlcN), galacturonic acid (GalA), galactosamine (GalN), glucose (Glc), and galactose (Gal) and the molar percentages of the monosaccharides are GlcN: 19.29%, GalA: 4.95%, GalN: 13.91%, Glc: 5.11%, and Gal: 56.73%. We investigated the AOS concentration, reaction time, and temperature using an orthogonal test and found that when the AOS concentration was 400 mg/mL and the reaction was conducted for half an hour at 60 °C. The particle size of SeNPs-AOS was 80 nm determined using a scanning electron microscope (SEM), and the elemental characterization based on energy dispersive spectroscopy (EDS) showed that the Se content was 8% in the SeNPs-AOS [27].

### 2.2. Experimental Design, Birds, and Diet

A total of 192 21-day-old unsexed Arbor Acres broilers (CP group in Zhanjiang, Guangdong, China) with similar body weight were assigned to a 2 × 2 experimental design. These were as follows: the thermoneutral zone group (TN), thermoneutral zone + SeNPs-AOS group (TN + 5 mg/kg SeNPs-AOS), heat stress group (HS), and heat stress + SeNPs-AOS group (HS + 5 mg/kg SeNPs-AOS). The experimental groups had six replicates of eight chickens per replicate (48 birds/group) and maintained for 21 days of experimentation. The ambient temperature of the TN groups was maintained at 23 ± 1.5 °C. The ambient temperature of the HS groups from 8:00 a.m. to 6:00 p.m. was maintained at 33 ± 2 °C, and the rest time was the same as TN groups. Humidity was maintained between 60–75% in all treatment groups. The basal diet was prepared according to NRC (1994). Broilers in TN and HS groups were fed the basal diet, while broilers in the additive groups were fed basal diet contained 5 mg/kg SeNPs-AOS. The content of selenium in the diet of the control group was 0.282 mg/kg, and supplementation SeNPs-AOS group was 0.696 mg/kg. Basal diet and nutrient composition are presented in Table 1.

### 2.3. Sample Collection

At the end of feeding experiment (42-day old), one broiler was randomly selected from each replicate for neck bloodletting until death (*n* = 6/treatment). Broilers were individually weighed, and collected for spleen, bursa of Fabricius and liver. Then, samples in each group were placed in an enzyme-free tube with 4% formaldehyde solution and stored at room temperature for the preparation of tissue sections. In order to be used for subsequent antioxidant determination and gene expression analysis, the remaining samples were placed in a sterile frozen tube and quickly frozen with liquid nitrogen for further analysis.

### 2.4. Organs Index and Histological Analysis

Complete spleens, bursae of Fabricius, and livers were taken from the broilers, then weighed and recorded to calculate the organ index. The organ index of the spleens, bursae of Fabricius and livers were calculated using the following formula: = (organ weight (g)/body weight (g)) × 100%.

For histological analysis, the spleen, bursa of Fabricius and liver samples were fixed in 10% neutral-buffered formalin for 48 h. Then, the samples were stained with hematein and eosin (H&E) by Wuhan Sevier Biotechnology Co., LTD. (Wuhan, China). At last, the slides were observed with a fluorescence microscope (GD-30REL) under 200× and 400× magnification. The image was collected by the CapStudio software (Version No. 3.8.6).

### 2.5. Antioxidant Parameters

The kits were purchased from the Nanjing Jiancheng Institute of Biological Engineering (Nanjing, China), and the activities of catalase (CAT), glutathione peroxidase (GSH-Px), glutathione S-transferase (GST), total superoxide dismutase (T-SOD), the levels of malondialdehyde (MDA) and total antioxidant capacity (T-AOC) were determined using the corresponding kits following the specification. The units used for the antioxidant parameters are as follows: CAT; U/mg prot, GSH-Px; U/mg prot, GST; U/mg prot, T-SOD; U/mg prot, MDA; nmol/mg prot, T-AOC; and mmol/mg prot. In particular, the “U/mg prot” in CAT represents the amount of H_2_O_2_ broken down by 1 umol per second per mg of protein, the “U” in T-SOD is a SOD activity unit, which is the amount of SOD per mg of protein when the SOD inhibition rate reaches 50% in 1 mL of reaction solution, and the “mmol/g prot” in T-AOC indicates that when a sample at a certain concentration has the same inhibition rate as Trolox, divide the molar concentration of Trolox at that concentration by the protein concentration of the samples. The units used are the same as previous studies [28,29].

### 2.6. Quantitative Real-Time PCR Analysis

The total RNA was extracted from spleen, bursa of Fabricius and liver using 1 mL RNA lysate (Trizol reagent) followed the manufacturer’s instructions. Then, the total RNA was reversed into complementary DNA (cDNA) by referring to the reverse transcription kit instructions of Novizan (Nanjing, China). After the reaction, it can be directly used for quantification or stored in the refrigerator at −20 °C.

According to the mRNA sequences of chicken selenoproteins, antioxidant-related genes and ferroptosis-related genes in NCBI Gene bank. The primers were synthesized by Shanghai Sangon Biological Engineering Co., Ltd. (Shanghai, China). Previous studies have found that the expression of *β-actin* in different tissues of broilers is relatively stable [30], therefore, real-time quantitative PCR used *β-actin* as the internal reference gene. The 2^−ΔΔCT^ method was used to calculate the relative expression of the target gene. The qPCR instrument used was from Bio-Rad (Watford, UK). The sequence information of the primer is detailed in Table 2.

### 2.7. Statistical Analysis

Data were analyzed by 2 × 2 analysis of variance (ANOVA) using the general linear model (GLM) program from the SAS 9.4 software, and multiple comparisons were made using Tukey’s test. In this case, *p <* 0.05 indicates a significant difference, and 0.05 *≤ p <* 0.10 indicates that it tends to be significant.

## 3. Results

### 3.1. Spleen Index and Histological Analysis

**Figure 1:**
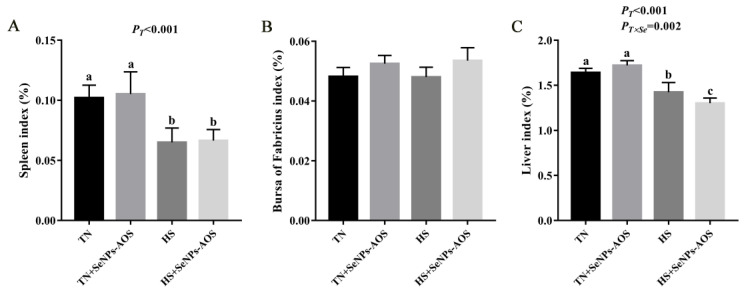
Effects of dietary biogenic selenium nanoparticles synthesized by alginate oligosaccharides (SeNPs-AOS) on spleen index (**A**), bursa of Fabricius index (**B**) and liver index (**C**) in broilers under heat stress. TN, thermoneutral zone group; TN + SeNPs-AOS, thermoneutral zone supplemented 5 mg/kg SeNPs-AOS; HS, heat stress group; HS + SeNPs-AOS, heat stress supplemented 5 mg/kg SeNPs-AOS. *P_T_*, main effect of ambient temperature *p* value; *P_Se_*, main effect of SeNPs-AOS *p* value; *P_T×Se_*, *p* values of interaction effect between ambient temperature and SeNPs-AOS. ^a,b,c^ Different superscript letters indicate significant differences (*p <* 0.05).

**Figure 2:**
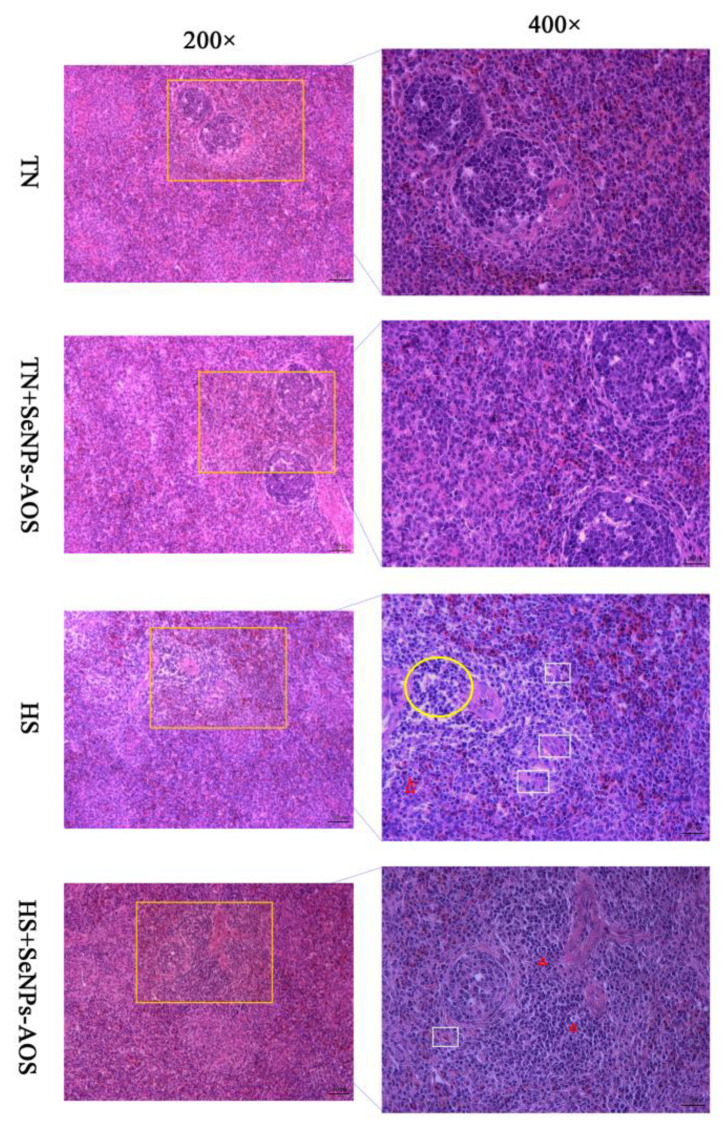
Effects of dietary biogenic selenium nanoparticles synthesized by alginate oligosaccharides (SeNPs-AOS) on the spleen morphological structure of heat-stressed broilers. TN, thermoneutral zone group; TN + SeNPs-AOS, thermoneutral zone supplemented 5 mg/kg SeNPs-AOS; HS, heat stress group; HS + SeNPs-AOS, heat stress supplemented 5 mg/kg SeNPs-AOS. Yellow oval represents the white pith merges with each other. Red triangle represents inflammatory cell infiltration. White square represents red pulp. The scar bar is 50 μm of 200× and 25 μm of 400×.

As shown in Figure 1A, HS reduced the spleen index (*p <* 0.01). The supplementation of SeNPs-AOS had no effect on spleen index in broilers under HS (*p >* 0.05). The spleen histological analysis (Figure 2) showed that HS decreased the number of spleen white pulps and had mutual fusion, increased the number of red pulps, and accompanied by a small number of inflammatory cells. Compared with the HS group, supplementation of SeNPs-AOS reduced spleen bleeding and a mass of red pulps.

### 3.2. Antioxidant Capacity and Relative mRNA Expression of Selenoprotein and Antioxidant-Related Genes in Spleen

**Figure 3:**
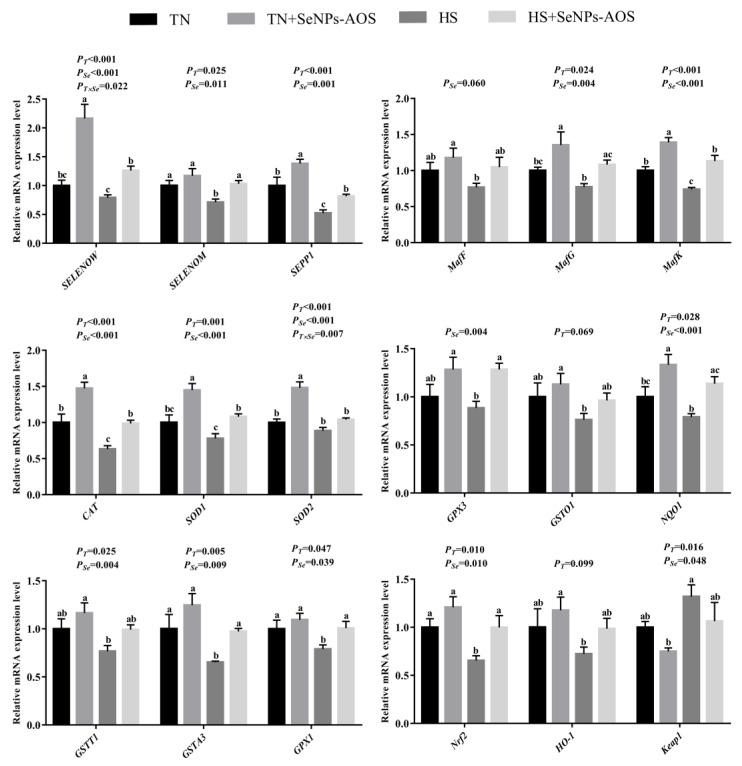
Effects of dietary biogenic selenium nanoparticles synthesized by alginate oligosaccharides (SeNPs-AOS) on relative mRNA expressions of selenoprotein and antioxidant-related genes of spleen in broilers under heat stress. TN, thermoneutral zone group; TN + SeNPs-AOS, thermoneutral zone supplemented 5 mg/kg SeNPs-AOS; HS, heat stress group; HS + SeNPs-AOS, heat stress supplemented 5 mg/kg SeNPs-AOS; *P_T_*, main effect of ambient temperature *p* value; *P_Se_*, main effect of SeNPs-AOS *p* value; and *P_T×Se_*, *p* values of interaction effect between ambient temperature and SeNPs-AOS. ^a,b,c^ Different superscript letters indicate significant differences (*p <* 0.05).

As depicted in Table 3, HS decreased the T-AOC (*p <* 0.05) level in the spleen, whereas increased MDA (*p <* 0.05) content was detected. Compared with the HS group, dietary SeNPs-AOS improved the activities of CAT (*p <* 0.001) and GSH-Px (*p <* 0.05) in the spleen. Results of the relative mRNA expression levels of selenoprotein and antioxidant-related genes are illustrated in Figure 3. The relative mRNA expression levels of *selenoprotein W* (*SELENOW*), *SEPP1*, *masculoaponeurotic fibrosarcoma K* (*MafK*), *CAT*, *SOD1*, *SOD2,* and *NAD (P)H: quinone oxidoreductase 1* (*NQO1*) genes in TN + SeNPs-AOS group were higher than TN group (*p <* 0.05). HS decreased *SELENOW*, *SEPP1*, *MafK*, *CAT*, *GSTA3*, *GPX1,* and *nuclear factor (erythroid-derived-2)-like 2* (*Nrf2*) mRNA relative expression levels (*p <* 0.05). Compared with the HS group, supplementation of SeNPs-AOS increased the relative mRNA expression levels of *the SELENOW*, *SEPP1*, *MafG*, *MafK*, *CAT*, *SOD1*, *GPX3*, *NQO1*, *GSTA3*, *GPX1,* and *Nrf2* genes (*p <* 0.05).

### 3.3. Bursa of Fabricius Index and Histological Analysis

**Figure 4:**
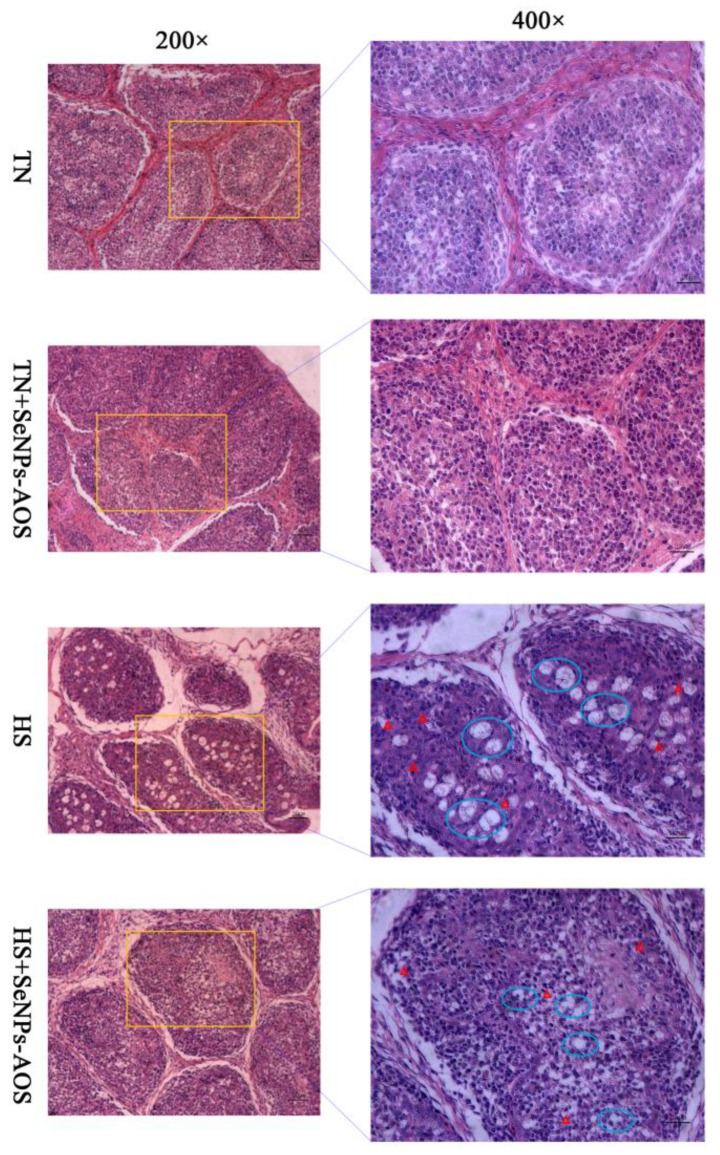
Effects of dietary biogenic selenium nanoparticles synthesized by alginate oligosaccharides (SeNPs-AOS) on the bursa of Fabricius morphological structure of heat-stressed broilers. TN, thermoneutral zone group; TN + SeNPs-AOS, thermoneutral zone supplemented 5 mg/kg SeNPs-AOS; HS, heat stress group; and HS + SeNPs-AOS, heat stress supplemented 5 mg/kg SeNPs-AOS. The blue ovals represent the vacuole. Red triangles represents inflammatory cell infiltration. The scar bar is 50 μm of 200× and 25 μm of 400×.

As presented in Figure 1B, HS and SeNPs-AOS supplementation had no significant effect on the bursa index (*p >* 0.05). As shown in Figure 4, HS caused the boundary between the medulla and cortex in the bursa to be blurred compared to the TN group. In addition, in the HS group the number of lymphocytes decreased and the volume reduced in the bursa and a large number of vacuoles appeared in the tissue. However, the supplementation of SeNPs-AOS decreased the number of hollow vesicles in the bursa increased the number of lymphocytes and arrangement relatively tightened.

### 3.4. Antioxidant Capacity and Relative mRNA Expression of Selenoprotein and Antioxidant-Related Genes in Bursa of Fabricius

**Figure 5:**
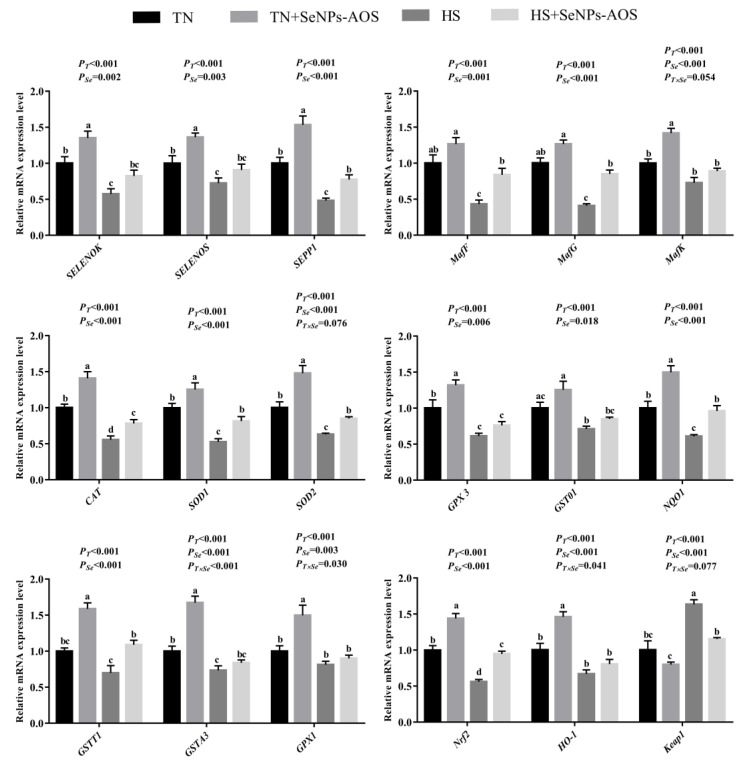
Effects of dietary biogenic selenium nanoparticles synthesized by alginate oligosaccharides (SeNPs-AOS) on relative mRNA expression of selenoprotein and antioxidant-related genes of bursa of Fabricius in broilers under heat stress. TN, thermoneutral zone group; TN + SeNPs-AOS, thermoneutral zone supplemented 5 mg/kg SeNPs-AOS; HS, heat stress group; HS + SeNPs-AOS, heat stress supplemented 5 mg/kg SeNPs-AOS; *P_T_*, main effect of ambient temperature *p* value; and *P_Se_*, main effect of SeNPs-AOS *p* value; *P_T×Se_*, *p* values of interaction effect between ambient temperature and SeNPs-AOS. ^a,b,c^ Different superscript letters indicate significant differences (*p <* 0.05).

The effects of dietary SeNPs-AOS on the antioxidant capacity of heat-stressed broilers are presented in Table 4. The activities of GST, GSH-Px, T-SOD and the level of T-AOC (*p <* 0.05) were decreased, and the content of MDA (*p <* 0.001) was increased by HS. Compared to the HS group, the supplementation of SeNPs-AOS increased the activities of GST, GSH-Px, CAT, and the level of T-AOC (*p <* 0.05) and declined the content of MDA (*p <* 0.001). Figure 5 shows the results of genes expression in bursa. The relative mRNA expression levels of *SEPP1*, *MafK*, *CAT*, *GSTT1*, *GSTA3*, *GPX1*, *Nrf2,* and *HO-1* in TN + SeNPs-AOS group were higher than TN group (*p <* 0.05). The expression of *SELENOK*, *SELENOS*, *SEPP1*, *MafF*, *MafG*, *CAT*, *SOD1*, *GPX3*, *GSTO1*, *NQO1*, *GSTA3,* and *Nrf2* genes were decreased by HS (*p <* 0.05), and the mRNA relative expression level of *Kelch-like ECH-associated protein 1* (*Keap1*) was increased by HS (*p <* 0.05). Compared to the HS group, dietary SeNPs-AOS improved the relative mRNA expression levels of *SEPP1*, *MafG*, *SOD1*, *GSTT1,* and *Nrf2* (*p <* 0.05) and decreased the relative mRNA expression level of *Keap1 (p <* 0.05).

### 3.5. Liver Index and Histological Analysis

**Figure 6:**
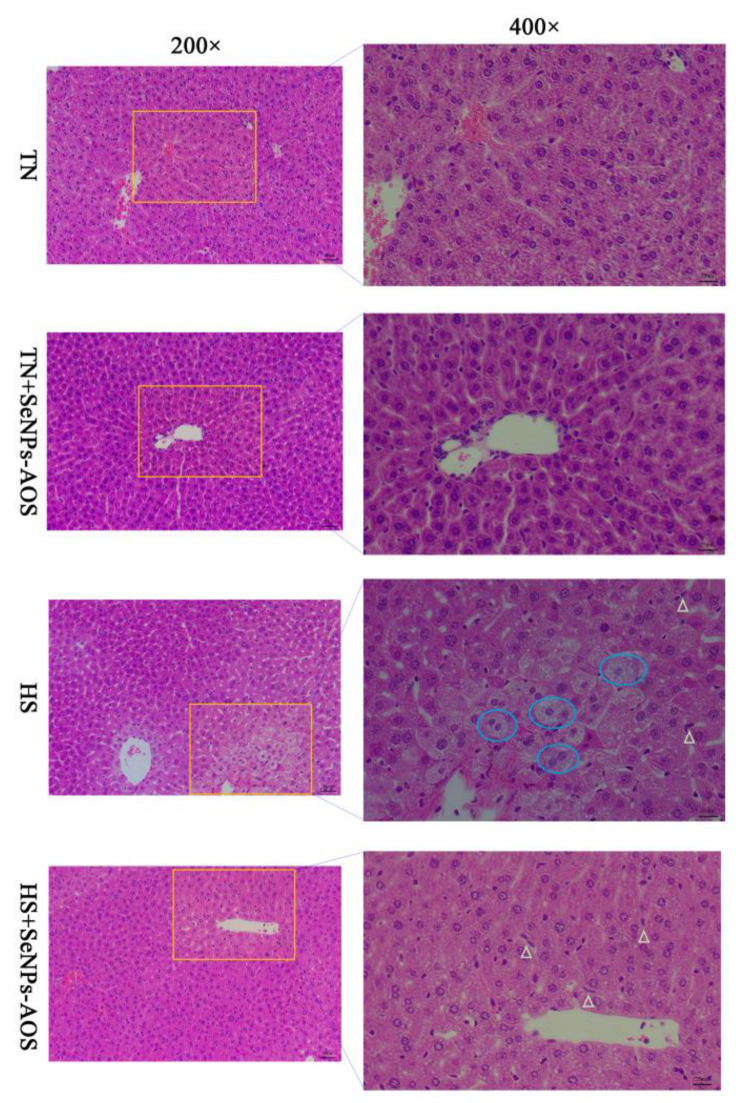
Effects of dietary biogenic selenium nanoparticles synthesized by alginate oligosaccharides (SeNPs-AOS) on the liver morphological structure of heat-stressed broilers. TN, thermoneutral zone group; TN + SeNPs-AOS, thermoneutral zone supplemented 5 mg/kg SeNPs-AOS; HS, heat stress group; and HS + SeNPs-AOS, heat stress supplemented 5 mg/kg SeNPs-AOS. The blue ovals represent hpatocyte ballooning; white triangles represent inflammatory cell infiltration. The scar bar is 50 μm of 200× and 25 μm of 400×.

As indicated in Figure 1C, broilers in HS groups had reduced the relative weight of the liver (*p <* 0.05). As shown in Figure 6, histological analysis of the liver showed that there were no pathological changes in the TN group. HS caused liver cells to swell and balloon, and some liver cells had inflammatory cell infiltration. In contrast, compared with the HS group, SeNPs-AOS supplementation could improve the pathological conditions of liver cell necrosis and swelling.

### 3.6. Antioxidant Capacity and Relative mRNA Expression of Selenoprotein and Antioxidant-Related Genes in Liver

**Figure 7:**
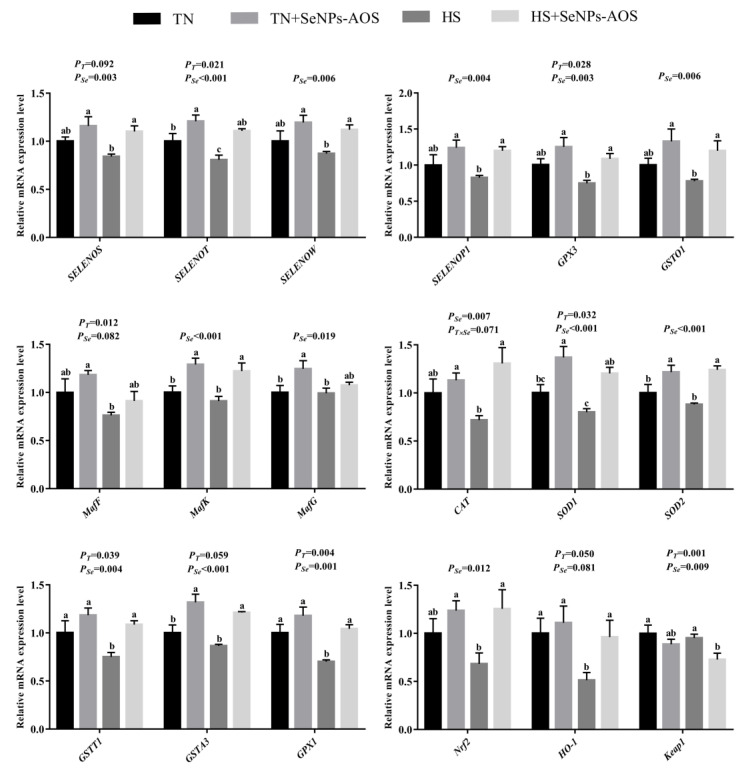
Effects of dietary biogenic selenium nanoparticles synthesized by alginate oligosaccharides (SeNPs-AOS) on relative mRNA expression of selenoprotein and antioxidant-related genes of liver in broilers under heat stress. TN, thermoneutral zone group; TN + SeNPs-AOS, thermoneutral zone supplemented 5 mg/kg SeNPs-AOS; HS, heat stress group; HS + SeNPs-AOS, heat stress supplemented 5 mg/kg SeNPs-AOS. *P_T_*, main effect of ambient temperature *p* value; *P_Se_*, main effect of SeNPs-AOS *p* value; and *P_T×Se_*, *p* values of interaction effect between ambient temperature and SeNPs-AOS. ^a,b,c^ Different superscript letters indicate significant differences (*p <* 0.05).

The results of hepatic antioxidant performance in broilers are presented in Table 5. HS decreased the activity of GSH-Px but increased the content of MDA (*p <* 0.001), whereas supplementation of SeNPs-AOS improved the activities of GSH-Px and GST and decreased the content of MDA (*p <* 0.05). As shown in Figure 7, the relative mRNA expression levels of *SELENOT*, *MafK*, *MafG*, *SOD1*, *SOD2,* and *GSTA3* in TN + SeNPs-AOS group were higher than TN group (*p <* 0.05). HS reduced the relative mRNA expression of *SELENOT*, *GSTT1*, *Nrf2,* and *GPX1* genes (*p <* 0.05), while dietary SeNPs-AOS upregulated the relative mRNA expression of *SELENOS*, *SELENOT*, *SELENOW*, *SELENOP1*, *Nrf2*, *CAT*, *SOD1*, *SOD2*, *GSTA3*, *GPX1*, *GPX3,* and *GSTO1* (*p <* 0.05).

### 3.7. Relative mRNA Expression of Ferroptosis-Related Genes

**Figure 8:**
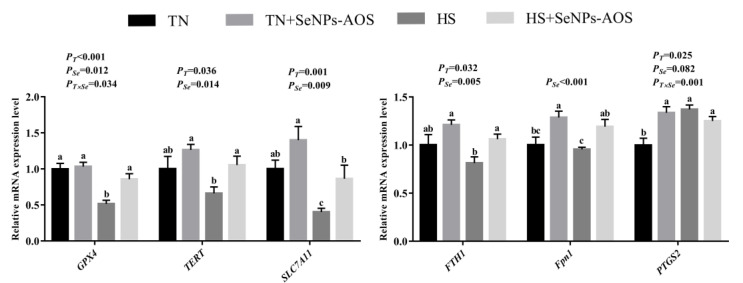
Effects of dietary biogenic selenium nanoparticles synthesized by alginate oligosaccharides (SeNPs-AOS) on relative mRNA expression of ferroptosis-related genes of liver in broilers under heat stress. TN, thermoneutral zone group; TN + SeNPs-AOS, thermoneutral zone supplemented 5 mg/kg SeNPs-AOS; HS, heat stress group; and HS + SeNPs-AOS, heat stress supplemented 5 mg/kg SeNPs-AOS. *P_T_*, main effect of ambient temperature *p* value; *P_Se_*, main effect of SeNPs-AOS *p* value; and *P_T×Se_*, *p* values of interaction effect between ambient temperature and SeNPs-AOS. ^a,b,c^ Different superscript letters indicate significant differences (*p <* 0.05).

As illustrated in Figure 8, HS downregulated the mRNA expression of *GPX4*, Texas emergency response team (*TERT*), solute carrier family 7 member 11 (*SLC7A11*), ferritin heavy polypeptide-1 (*FTH1*), and ferroportin 1 (*Fpn1*) (*p* < 0.05) and increased the mRNA expression of post-transcriptional gene silencing-2 (*PTGS2*) (*p* < 0.05). In contrast, the supplementation of SeNPs-AOS upregulated the mRNA expression levels of *GPX4, TERT, SLC7A11, FTH1*, and *Fpn1* (*p* < 0.05).

## 4. Discussion

### 4.1. Effects of Dietary SeNPs-AOS Supplementation on the Spleen of Heat-Stressed Broilers

The spleen is an important peripheral immune organ for broilers. An increase in spleen weight represents an enhancement of splenic function, thereby enhancing the tolerance to oxidative damage [31]. It has been demonstrated that HS inhibited the development of the spleen and even caused the splenic damage [32]. As the HS time increases, an enema occurs in the spleen tissue [33]. The results of this study are consistent with previous studies, which found that HS decreased the relative weight of spleen and caused the spleen pulp to fuse together and the number of white pulps to decrease, whereas the number of red pulps were increased and accompanied by a small number of inflammatory cells in the spleen [34]. In our previous trial, supplementation with 5 mg/kg SeNPs-AOS had a significant anti-HS effect through in vivo experiment [27]. Interestingly, dietary 5 mg/kg SeNPs-AOS supplementation reduced spleen bleeding and a mass of red pulps, protected the normal morphological structure of the spleen. It has been reported that natural polymers play a key role in the formation and enhancement of the SeNPs stability, the AOS was used as the polymer template in this experiment, and the hydrogen bonds between AOS molecules were used to interact with the surface of SeNPs, which provides higher surface activity and reduction ability to the SeNPs, thereby Se could better exert its antioxidant capacity [35,36]. Alian et al. [37] suggested that SeNPs could not only increase the spleen index, but also improve the antioxidant status of broilers. Several studies have shown that dietary Se could alleviate injuries described histomorphologically that tissue lipid oxidation and inflammatory cell infiltration in heat-stressed broilers [38,39]. We speculated that the reason for improving this damage may be due to the combined antioxidant effect of AOS and Se. Also, the high surface activity and nanoscale effects enable biogenic SeNPs to have good organ protection efficiency [24], so we previously observed that SeNPs-AOS improved growth performance of heat-stressed broilers [27]. However, in the future, further determinations of inflammatory cytokine-related indicators should be conducted to better explain the role of SeNPs-AOS.

Free radicals produced by HS led to a suppression of antioxidant enzymes activity, thus destroying the structure and function of organs [40]. The antioxidant enzymes include CAT, SOD, GST, GSH-Px, etc. SOD is an important antioxidant enzyme in animals, which can clear the superoxide anion free radicals and protect cells from damaging [41,42]. T-AOC is a crucial parameter to evaluate the overall antioxidant status of cells [43]. Notably, Se usually plays its physiological role in the form of selenoproteins, the proteins synthesized using selenocysteines [44]. Relevant studies have shown that selenoproteins could drive the body’s biological reaction processes to enhance antioxidant ability in broilers [45,46]. Among them, it is worth noting that *SELENOM* participates in the synthesis of disulfide bonds, increases the activity of GSH-Px, and maintains redox homeostasis [47]. As we know, Nrf2 signaling pathway is the main antioxidant pathway, but Keap1 inhibits the expression of Nrf2 protein [48]. In addition, Nrf2 and Maf proteins can form dimers, which subsequently bind to Maf-recognizing elements, thereby inducing Maf proteins to encode antioxidant enzymes, and the relative expression of *Nrf2* and *Maf* increased, the body’s antioxidant capacity is enhanced [49]. Consistent with previous reports, HS resulted in a decrease in the activity of antioxidant enzymes and the downregulation of *Nrf2* and antioxidant-related genes in the spleen. It is interesting that SeNPs-AOS has exerted superior antioxidant effects in our study. The specific performances of SeNPs-AOS were the improved activities of CAT and GSH-Px, and the elevated mRNA expression of the *SELENOW*, *SEPP1*, *MafG*, *MafK*, *CAT*, *SOD1*, *GPX3*, *NQO1,* and *Nrf2* genes. Similarly, earlier studies have shown that dietary supplementation of SeNPs effectively ameliorated the adverse effects of oxidative stress [50,51]. Hence, it is reasonable to speculate that SeNPs-AOS could activate the Nrf2 pathway to mitigate HS-induced oxidative damage in spleen.

### 4.2. Effects of Dietary SeNPs-AOS Supplementation on the Bursa of Heat-Stressed Broilers

The bursa is an immune organ unique to birds and it is pivotal for broilers [52]. The development of the bursa is closely related to lymphocytes. It has been documented that the destruction of lymphocytes leads to the deficiency of bursal function [53]. Numerous studies have shown that HS inhibited the production of antibodies while increasing the expression of inflammatory factor genes, resulting in a compromised immune system and an inhibition of antioxidant ability [54,55]. HS reduces the follicular area of the bursa vessel, the structure is loose and disordered, and vacuoles appear in the medulla [56]. Also, previous a study suggested that HS led to pathological changes, lymphocyte necrosis, cellular structure injury, and extensive infiltration of inflammatory cells in the bursa of broilers [57]. The above studies are in accordance with the present results, and we found that HS caused morphological lesions of bursa in broilers. Unfortunately, there was no statistical difference in the organ index of bursa of Fabricius in broilers under the conditions of this experiment. Liu et al. [57] and Hosseini et al. [58] also found that HS had no significant effect on the organ index of bursa of Fabricius in broilers. Furthermore, this study observed that SeNPs-AOS supplementation could improve the bursal damage of heat-stressed broilers, and the vacuolization phenomenon and inflammatory cell infiltration were reduced by SeNPs-AOS. Similarly, Sun et al. [59] indicated that organic Se sources promoted the expression of various selenoproteins and maintained the integrity of immune organs. However, there is lack of researches on the protective effect of biogenic SeNPs on poultry bursa currently, so direct comparison is incapable. Such beneficial roles of SeNPs-AOS may be associated with the regulation of antioxidant related selenoproteins synthesis in bursa. It should be noted that more research is needed on the expression of immune-related genes to further reveal the mechanism of SeNPs-AOS in alleviating HS-induced impairment of the bursa.

HS causes an imbalance between oxidation and antioxidant defense system, and leads to the body to produce a large amount of ROS, whereas excess ROS can damage cells and tissues, ultimately resulting in serious oxidative damage to organs [60]. The ability of scavenging free radicals is mainly determined by the activities of antioxidant enzymes such as GST, while the content of MDA reflects the degree of lipid peroxidation [61]. We found that HS led to low activities of antioxidant enzyme in bursa and reduced the expression of antioxidant-related genes, which indicated that the bursal damage was occurred by oxidative stress, this is similar to the results by Truong et al. [62] and Li et al. [63]. GSH-Px is known for the antioxidant function, while Se directly affects its activity [64]. In addition, Se exerts a variety of antioxidant functions by regulating the synthesis of multiple antioxidant enzymes and glutathione, and participates in antioxidant biological pathways in vivo [17]. Specifically, Se can be converted to hydrogen selenide for further Se-cystine synthesis, which provides cysteine to glutathione and allow it to exert its antioxidant function [65]. Past studies have revealed that selenoproteins could be involved in biological responses such as antioxidant, anti-inflammatory, etc. [46]. For instance, SELENOK reduces ROS activity in mouse cardiomyocytes and resists hydrogen peroxide (H_2_O_2_)-induced cytotoxicity [66]. Aligning with this, dietary SeNPs-AOS enhanced enzyme activities of GST, GSH-PX, and CAT possibly through promoting the synthesis of antioxidant selenoproteins, such as *SEPP1* and *GSTT1*, thus easing the oxidative damage to the bursa caused by HS.

### 4.3. Effects of Dietary SeNPs-AOS Supplementation on the Liver of Heat-Stressed Broilers

The liver is a key metabolic organ, the abnormal liver index and histopathological changes result in hepatic dysfunction, which negatively affects nutrient metabolism and animal health [67]. Under HS conditions, pathological changes will occur in the liver, including cell necrosis and structure destruction, inflammatory cell infiltration, and lipid vacuolation [68,69]. It has been confirmed that the liver is more vulnerable to damage than other organs during HS [70]. Consistently, we found that HS reduced the relative weight and caused morphological damage of liver in broilers. Dietary antioxidants were reported to improve the liver function in broilers under HS [50]. Previous studies have also demonstrated that supplementation of SeMet could promote liver function [71]. It has been shown that dietary Se could reverse liver damage, and alleviated oxidative stress in hepatocytes via regulating Keap1/Nrf2 pathway [72]. In the present study, SeNPs-AOS supplementation relieved the pathological conditions of liver by reducing cell necrosis and swelling. Suggesting that dietary SeNPs-AOS is beneficial for the liver health of broilers raised in a thermal environment. In addition, we have previously found that dietary SeNPs-AOS could improve the growth performance of heat-stressed broilers, this may also be attributed to the relieving effect of SeNPs-AOS on liver injury [27].

To confirm the restorative effect of SeNPs-AOS on hepatic injury in heat-stressed broilers, we determined the antioxidant performance and expression of related signaling molecules in the liver. The current findings showed that HS reduced GSH-Px activity and increased MDA content, and downregulated the expression of *SELENOT*, *GSTT1*, *GPX1* and *HO-1* genes of liver. Actually, numerous studies have indicated that HS suppressed the antioxidant enzymes activities in liver and other organs [73,74]. Cao et al. [75] and Sahin et al. [76] found that HS resulted in a decline in mRNA expression level of *Nrf2*, *SOD*, *GST*, *HO-1*, etc. Conversely, previous studies showed that selenomethionine supplements enhanced the level of T-AOC and the ability of T-SOD in H_2_O_2_-induced liver damage model [77]. Wan et al. [78] verified that selenomethionine could improve the glutathione system to enhance the antioxidant capacity of liver. It’s worth noting that, in this study, dietary SeNPs-AOS improved the antioxidant enzymes activities and upregulated the mRNA expression levels of *SELENOS*, *SELENOT*, *SELENOW*, *SELENOP1*, *GPX3*, *GSTO1*, *MafK*, *CAT*, *SOD1*, *SOD2*, *GSTT1*, *GSTA3*, *GPX1*, *HO-1,* and *Nrf2* genes in liver of heat-stressed broilers. This is consistent with previous literature that supplementation of Se could upregulate the mRNA levels of *Nrf2* and its downstream genes (*NQO1* and *HO-1*) to mitigate oxidative stress caused by excess H_2_O_2_ [79]. Meanwhile, we noticed that oligosaccharides also have antioxidant functions. Unsurprisingly, Liu et al. [28] found that oligosaccharides could act as free radical scavenger according to their chemical structure, and exert antioxidant function. Therefore, Se and AOS may both contribute to SeNPs-AOS in alleviating hepatic oxidative stress, and, unlike traditional forms of selenium, its unique nanoscale effect may enhance the protective effect.

Ferroptosis is caused by a large accumulation of lipid peroxides, which is also an important adverse consequence of oxidative stress and can exacerbate organ damage [80]. Wei et al. [81] have demonstrated that ferroptosis usually occurs in hepatocytes under oxidative conditions, because oxidative stress inhibited the expression of anti-ferroptosis genes, thus inducing more severe hepatic damage. In this study, HS decreased the mRNA expression of *GPX4* and *SLC7A11*, and increased the expression of *PTGS2* gene, which is consistent with previous studies [82]. It is well known that GPX4 is one of the key regulators of ferroptosis, and when GPX4 is inactivated, it triggers the accumulation of lipid peroxides [83]. SLC7A11 is one of the most critical upstream regulators of ferroptosis and able to enhance the anti-ferroptosis activity of GPX4, and Nrf2 can bind to the promoter regions of GPX4 and SLC7A11 to relieve ferroptosis [84]. Moreover, FTH1 and TERT protect cells from the effects of GPX4 inhibitors. As an iron transporter, Fpn1 can alleviate ferroptosis by reducing the excessive accumulation of Fe^2+^ in the cells [85]. Previously, Zhao et al. [86] have shown that supplementation of Se reduced ferroptosis in heart cells. It has been found that dietary Se could activate the Nrf2/SLC7A11/GPX4 signaling pathway to suppress ferroptosis [87,88]. Similarly, as shown in present study, dietary SeNPs-AOS upregulated the relative mRNA expression levels of *GPX4*, *SLC7A11*, *TERT*, *FTH1,* and *Fpn1* in the liver of broilers under HS. Indicating that SeNPs-AOS could not only alleviate hepatic oxidative stress through Nrf2-mediated antioxidant pathway, but also upregulate the expression of anti-ferroptosis molecules that related to Nrf2/SLC7A11/GPX4 and transferrin pathways. However, this experimental bottleneck is that it has neither Prussian blue staining with tissues nor intracellular iron accumulation trial, so the anti-ferroptosis effect of SeNPs-AOS is still needed further research and verification.

## 5. Conclusions

In summary, we prepared the biogenic SeNPs-AOS to have good antioxidant activity in broilers, which was specifically manifested in alleviating oxidative damage of organs caused by HS, upregulating the expression of antioxidant-related genes and downregulating the expression of *keap1* gene, this is association with the activation of Nrf2 signaling pathway and the promotion of selenoproteins synthesis. At the same time, dietary SeNPs-AOS could upregulate the expression of anti-ferroptosis related genes belonging to Nrf2/SLC7A11/GPX4 and transferrin pathways. These findings not only provide a foundation for further reference on the anti-HS of biogenic SeNPs in broilers, but also offer novel insights into studying SeNPs-AOS as a new type of antioxidants to protect organs from oxidative damage.

## Figures and Tables

**Table 1:** Basal diet composition and ingredients.

Item	Contents (%)
Ingredients	
Corn	55.00
Soybean meal	34.82
Wheat bran	2.00
Soybean oil	5.00
Limestone	0.50
CaHPO4	1.60
NaCl	0.30
DL-Methionine	0.18
L-Lysine (50%)	0.10
Vitamin Premix ^1^	0.20
Mineral premix ^2^	0.30
total	100
Nutrient levels	
ME (MJ/kg)	12.82
Crude protein (%)	19.92
Ca (%)	0.93
P (%)	0.44
Lys (%)	1.30
Met (%)	0.45
Total Met + Cystine (%)	0.72
Se (%) ^3^	Measured value

^1^ Premix provided per kilogram of diet: 9000 IU of vitamin A, 3240 IU of vitamin D3, 6 IU of vitamin E, 30.75 mg of vitamin K, 1.5 mg of vitamin B1, 4.5 mg of vitamin B2, 1.5 mg of vitamin B6, 10 μg of vitamin B12, 9 mg of niacin, 0.45 mg of folic acid, 1000 mg of choline, and 9 mg of pantothenic acid. ^2^ Premix provided per kilogram of diet: 40 mg of Zn, 60 mg of Mn, 80 mg of Fe, 8 mg of Cu, 0.35 mg of I, 0.15 mg of Se. ^3^ Se was the mean value of three repeated tests of experimental diet samples. The content of Se in the experimental diet of the control group was 0.282 mg/kg, and that of the SeNPs-AOS group was 0.696 mg/kg.

**Table 2:** Primers used for quantitative real-time PCR.

Target Genes	Primer	Primer Sequence (5′→3′)	Accession No.
*SELENOM*	Forward	GGCTTCTACCGCAAGGAGACTC	NM_001277859.2
	Reverse	GGTGGTCCTTCTTGTCCTGTTCA	
*SELENOW*	Forward	CAGGAGGTGACGGGATGGTT	NM_001166327.2
	Reverse	TACGGGAGGGCAGCTTGGAT	
*SELENOS*	Forward	CGTCGCCATCTATCTCATCGT	NM_001024734.3
	Reverse	GCTTCTTGTCTTCTTACCACCAT	
*SELENOT*	Forward	GGCACATAGCATCCTTCCTG	NM_001006557.4
	Reverse	CCGTTGACATACACTGGTTCT	
*SELENOP1*	Forward	CCAAGTGGTCATTCACATC	NM_001031609.2
	Reverse	ATGACGACCACCCTCACGAT	
*SEPP1*	Forward	GCAGACAGCATCAGACTCTACAAC	NM_001031609.3
	Reverse	TCAGGCAGCAGTGAGCAGAC	
*MafF*	Forward	CGACGACGGACGCTGAAGAA	NM_204757.2
	Reverse	GTACTTGCCACGGAGAGTGTCAA	
*MafK*	Forward	GCAGCAAGAGGTGGAGAAGC	NM_204756.2
	Reverse	ACGGCACGGAACTGGATGA	
*MafG*	Forward	ACGCTGAAGAACCGAGGCTAC	NM_001079489.1
	Reverse	GTTCTGGCGAAGTTCTGGAGTG	
*CAT*	Forward	CTATCCTTCCTGGTCTTTCTACAT	NM_001031215.2
	Reverse	TCATACGCCATCTGTTCTACCT	
*SOD1*	Forward	AGGGAGGAGTGGCAGAAGT	NM_205064.1
	Reverse	GCTAAACGAGGTCCAGCAT	
*NQO1*	Forward	ACCATCTCTGACCTCTACGCCATA	NM_001277619.1
	Reverse	GCCGCTTCAATCTTCTTCTGCTC	
*SOD2*	Forward	TCCTGACCTGCCTTACGACTATGG	NM_204211.1
	Reverse	GCGACACCTGAGCTGTAACATCAC	
*GPX1*	Forward	CAAAGTGCTGCTGGTGGTCAAC	NM_001277853.2
	Reverse	TTGGTGGCGTTCTCCTGGTG	
*GPX3*	Forward	TGGCAGAGGAGTTCGGCAAC	NM_001163232.2
	Reverse	CGTTCTTGACAGTGGCGATGTT	
*GSTT1*	Forward	CATGCTAACATCCGGGCTAA	NM_205365.1
	Reverse	AAATTGCTTCAGGGAAGTGG	
*GSTA3*	Forward	GCGGCTGCTGGAGTTGAGTT	NM_001001777.1
	Reverse	GTAGTTGAGGATGGCTCTGGTCTG	
*GSTO1*	Forward	GGGCTGGTTCCTGTTCTG	NM_001277375.1
	Reverse	TCTTCTGTAAGGCTCGCTCAT	
*Nrf2*	Forward	TGTGTGTGATTCAACCCGACT	NM_205117.1
	Reverse	TTAATGGAAGCCGCACCACT	
*Keap1*	Forward	ACTTCGCTGAGGTCTCCAAG	NM_012289.4
	Reverse	CAGTCGTACTGCACCCAGTT	
*HO-1*	Forward	GCCTACACCCGCTATTTGG	NM_205344.1
	Reverse	TCTCAAGGGCATTCATTCG	
*TERT*	Forward	TTCCTCGCTCCTCCCTCAGT	NM_001031007.2
	Reverse	CGGCATTTGTTATGGCTTGAACC	
*FTH1*	Forward	GAATGTGAACCAGTCGCTGTTAGA	NM_205086.2
	Reverse	AGGTACTCTGCCATGCCATACTT	
*Fpn1*	Forward	CCACAGCGATCACAATTCAGAGG	NM_001012913.2
	Reverse	CGACATCAGGTTCCAGCCAGAA	
*PTGS2*	Forward	TGGTGAGACTCTGGAGAGGCAACT	NM_001167718.2
	Reverse	GCCAAACACCTCCTGCCCAACA	
*GPX4*	Forward	ACCCGCTGTGGAAGTGGATGAAG	NM_001039848.4
	Reverse	TCACCACGCAGCCGTTCTTGT	
*β-actin*	Forward	GTGATGGACTCTGGTGATGGTGTT	NM_205518.1
	Reverse	TCTCGGCTGTGGTGGTGAAG	

**Table 3:** Effects of dietary biogenic selenium nanoparticles synthesized by alginate oligosaccharides (SeNPs-AOS) on spleen antioxidant performance of broilers under heat stress.

Parameters	TN		HS			*p*-Values		
	CON	SeNPs-AOS	CON	SeNPs-AOS	SEM	Temp.	SeNPs-AOS	Temp. × SeNPs-AOS
CAT, U/mg prot	7.64 ^b^	20.53 ^a^	7.21 ^b^	18.93 ^a^	0.85	0.249	<0.001	0.500
GSH-Px, U/mg prot	122.29 ^bc^	147.78 ^a^	108.26 ^c^	136.73 ^ab^	6.28	0.060	0.001	0.815
GST, U/mg prot	246.15 ^ab^	296.24 ^a^	218.66 ^b^	223.28 ^b^	19.99	0.021	0.186	0.269
T-SOD, U/mg prot	683.90 ^ac^	739.77 ^a^	602.47 ^c^	610.83 ^c^	28.67	0.002	0.276	0.417
T-AOC, mmol/g prot	0.92 ^c^	0.98 ^a^	0.94 ^b^	0.94 ^b^	0.005	0.290	<0.001	<0.001
MDA, nmol/mg prot	5.66 ^bc^	3.24 ^c^	9.39 ^a^	7.07 ^ab^	1.16	0.004	0.054	0.965

TN, thermoneutral zone; HS, heat stress; CON, control group, basal diet without addition of SeNPs-AOS; SeNPs-AOS, basal diet with addition of 5 mg/kg SeNPs-AOS; SEM, standard error of the mean; CAT, catalase activities; GSH-Px, glutathione peroxidase activities; GST, glutathione S-transferase activities; T-SOD, total superoxide dismutase activities; T-AOC, total antioxidant capacity; MDA, malondialdehyde. *p* value of Temp., main effect of temperature *p* value; *p* value of SeNPs-AOS, main effect of SeNPs-AOS *p* value; *p* value of Temp. × SeNPs-AOS, *p* values of interaction effect between temperature; and SeNPs-AOS. ^a,b,c^ means assigned different lowercase superscript letters are obviously different, *p <* 0.05.

**Table 4:** Effects of dietary biogenic selenium nanoparticles synthesized by alginate oligosaccharides (SeNPs-AOS) on the bursa of Fabricius antioxidant performance of broilers under heat stress.

Parameters	TN		HS			*p*-Values		
	CON	SeNPs-AOS	CON	SeNPs-AOS	SEM	Temp.	SeNPs-AOS	Temp. × SeNPs-AOS
CAT, U/mg prot	21.48 ^b^	57.92 ^a^	10.50 ^b^	53.13 ^a^	5.45	0.163	<0.001	0.504
GSH-Px, U/mg prot	18.30 ^b^	21.66 ^a^	8.73 ^c^	18.43 ^b^	1.02	<0.001	<0.001	0.005
GST, U/mg prot	173.60 ^b^	255.35 ^a^	82.82 ^c^	124.43 ^d^	12.52	<0.001	<0.001	<0.001
T-SOD, U/mg prot	754.94 ^b^	1240.35 ^a^	556.54 ^c^	618.32 ^bc^	58.36	<0.001	<0.001	<0.001
T-AOC, mmol/g prot	1.00 ^a^	1.01 ^a^	0.75 ^b^	0.97 ^a^	0.04	0.009	0.025	<0.001
MDA, nmol/mg prot	13.21 ^b^	17.18 ^a^	16.04 ^a^	12.52 ^b^	0.96	<0.001	<0.001	<0.001

TN, thermoneutral zone; HS, heat stress; CON, control group, basal diet without addition of SeNPs-AOS; SeNPs-AOS, basal diet with addition of 5 mg/kg SeNPs-AOS; SEM, standard error of the mean; CAT, catalase activities; GSH-Px, glutathione peroxidase activities; GST, glutathione S-transferase activities; T-SOD, total superoxide dismutase activities; T-AOC, total antioxidant capacity; MDA, malondialdehyde. *P* value of Temp., main effect of temperature *p* value; *p* value of SeNPs-AOS, main effect of SeNPs-AOS *p* value; and *p* value of Temp. × SeNPs-AOS, *p* values of interaction effect between temperature and SeNPs-AOS. ^a,b,c,d^ means assigned different lowercase superscript letters are obviously different, *p <* 0.05.

**Table 5:** Effects of dietary biogenic selenium nanoparticles synthesized by alginate oligosaccharides (SeNPs-AOS) on liver antioxidant performance of broilers under heat stress.

Parameters	TN		HS			*p*-Values		
	CON	SeNPs-AOS	CON	SeNPs-AOS	SEM	Temp.	SeNPs-AOS	Temp. × SeNPs-AOS
CAT, U/mg prot	269.29 ^b^	361.38 ^a^	232.44 ^b^	276.01 ^b^	20.40	0.007	0.003	0.248
GSH-Px, U/mg prot	107.53 ^b^	127.50 ^a^	50.05 ^d^	71.59 ^c^	5.86	<0.001	0.002	0.895
GST, U/mg prot	97.30 ^ab^	106.20 ^a^	90.41 ^b^	103.26 ^a^	4.17	0.252	0.017	0.641
T-SOD, U/g prot	26.64 ^a^	23.97 ^ab^	22.27 ^bc^	24.64 ^ab^	1.43	0.211	0.916	0.094
T-AOC, mmol/g prot	481.74 ^bc^	498.36 ^ab^	455.15 ^c^	453.98 ^c^	15.22	0.030	0.617	0.565
MDA, nmol/mg prot	12.77 ^a^	10.72 ^b^	16.67 ^c^	14.61 ^a^	0.674	<0.001	0.006	0.996

TN, thermoneutral zone; HS, heat stress; CON, control group, basal diet without addition of SeNPs-AOS; SeNPs-AOS, basal diet with addition of 5 mg/kg SeNPs-AOS; SEM, standard error of the mean; CAT, catalase activities; GSH-Px, glutathione peroxidase activities; GST, glutathione S-transferase activities; T-SOD, total superoxide dismutase activities; T-AOC, total antioxidant capacity; MDA, malondialdehyde. *p* value of Temp., main effect of temperature *p* value; *p* value of SeNPs-AOS, main effect of SeNPs-AOS *p* value; *p* value of Temp. × SeNPs-AOS, *p* values of interaction effect between temperature and SeNPs-AOS. ^a,b,c,d^ means assigned different lowercase superscript letters are obviously different, *p <* 0.05.

## Data Availability

Data is contained within the article.
